# Significant others

**DOI:** 10.1186/2041-2223-3-21

**Published:** 2012-10-18

**Authors:** Mark A Jobling

**Affiliations:** 1 Department of Genetics, University of Leicester, University Road, Leicester, LE1 7RH, UK

## 

Sir Arthur Conan Doyle's immortal creation was, of course, the violin-playing, cocaine-addicted genius Sherlock Holmes; but the great detective had an extensive supporting cast: the dependable but oft-baffled Dr Watson, the long-suffering Mrs Hudson, and a host of lesser characters, some more contemptible than others.

In the very lower echelons was one adversary, described by Watson [1] as ‘…a little black man … with a great, misshapen head and a shock of tangled, dishevelled hair. His … thick lips were writhed back from his teeth, which grinned and chattered at us with a half animal fury.’ This frightening figure hails from the Andaman Islands, and represents a people ‘naturally hideous, … fierce, morose, and intractable’, who ‘may perhaps claim the distinction of being the smallest race upon this earth. … They have always been a terror to shipwrecked crews, braining the survivors with their stone-headed clubs, or shooting them with their poisoned arrows. These massacres are invariably concluded by a cannibal feast.’ In a thrilling nocturnal chase along the Thames, the detective and his medical sidekick narrowly avoid being eliminated by a poisoned dart, and their ghastly assailant ends up dead in the river, thanks to a dose of trusty English lead.

Conan Doyle’s description is inaccurate in at least three respects (the record-breaking small stature, the poison, and the cannibalism), and may seem ridiculously extreme, but it rests upon a long tradition of vilification, from Ptolemy to Marco Polo, the latter allegedly describing the alien beings of the Andaman Islands as having the heads of dogs.

The Andamans form a remote archipelago of some 300 islands in the Indian Ocean, and what is certain about their hunter-gatherer inhabitants is that they have lived there for a long time, and (as implied by Conan Doyle) have a tradition of resisting contact with outsiders. The success of this strategy has varied. Of the four existing groups, one, the Great Andamanese, has reduced 100-fold in size to only a few tens of individuals, largely due to the ravages of imported measles, influenza, and syphilis. Contact here was the most sustained, following the establishment of a large prison colony by the British in 1858. The Onge, who live on Little Andaman Island, have been left more or less in peace and number around 100 individuals. The few hundred Jarawa people make up the largest group, and survive in the forests and coastal areas of Great Andaman.

Much interest has been focused on a mysterious fourth group, the Sentinelese. Inhabiting the small island of North Sentinel, these people (whose numbers are unknown) are notorious for their hostility to incomers and their reluctance to engage in contact via the traditional route of gift-giving. Nineteenth-century administrators initiated the practice of either leaving gifts (often a pig, or coconuts) on the beach and waiting, or of capturing tribal members, then releasing them after a while, laden with the fascinating and useful products of the Victorian economy. For some groups, this achieved the desired aim of habituation and reduced hostility, but not for the Sentinelese. Google-image them, and what you see are the kinds of grainy images usually reserved for topless British royalty: telephoto pictures taken from a safe distance. Famously, a helicopter was sent to see if the islanders had survived the 2004 tsunami, and the robust answer to that question is immortalized in the image of a man firing skyward arrows from the ravaged beach.

It is often stated that the people of the Andaman Islands have lived there in isolation for as long as 30,000 or even 60,000 years, and represent an early outpost of the out-of-Africa migrations. Archaeological evidence is scanty, and so far provides confirmation of human presence for only the last 2,500 years or so; however, anthropologists believe the occupation is much more ancient. The languages of the islands are unrelated to any others, and according to Ethnologue (http://www.ethnologue.com) constitute one of the world’s 128 language families, though some linguists consider Jarawa-Onge to be a distinct family from Great Andamanese. Confusion exists about the language of the Sentinelese; while Ethnologue groups it with the other Andamanese languages, another observer [2] points out that no-one has apparently ever got close enough to these people to attempt a proper conversation with them, and proposes (by analogy to the long-lens capture of images) the use of sensitive directional microphones to record and analyze their conversations from boats offshore.

Physical anthropologists have long noted that the Andaman Islanders and other so-called ‘Negrito’ populations of Asia share a set of physical features, including short stature, very dark skin, and scanty body hair, with African pygmies. There is also a similarity in skull morphology, while the skulls of other Asian Negritos resemble those of Melanesians.

And what of the genetics? Unsurprisingly given the dismal colonial history, Y-chromosome analysis showed modern Great Andamanese to have undergone male-mediated admixture with Indians and East Asians. But the Y chromosomes of Jarawa and Onge belong to a deep-rooting lineage within an Asian haplogroup, D [3], and the mitochondrial DNAs of all groups to deep-rooting lineages within haplogroup M [3-5]. This rules out any recent relationship with Africa, and has been taken to be consistent with the Andaman Islanders representing an early offshoot of the out-of-Africa migration, about 50,000 years ago. Whole-genome SNP analysis of a wide range of Indian populations [6] confirms admixture in the history of the Great Andamanese, but shows the Onge to be the only studied population with 100% membership of an ‘Ancestral South Indian’ cluster, consistent with an early origin and a long history of isolation.

The 19th-century British administrators of the Islands regularly captured groups of Andamanese and dispatched them by boat to Calcutta, where they were at first housed, like animals, at the Zoological Gardens, and visited by the local populace. When a mother and child died there, their bodies were preserved in a large glass jar and stored with other specimens in the Calcutta Museum basement [7].

From our privileged position in the enlightened 21st century we might think such attitudes have vanished, but unfortunately it is not entirely so. In the late 1960s, the Indian government constructed the Andaman Trunk Road through the forests of the Jarawa, and although India’s Supreme Court ordered its closure 10 years ago, it remains very much open. These days it is a highway for air-conditioned tourist buses taking their customers on ‘human safaris’ [8], on which they hope to capture a video of semi-naked Jarawa women, who are urged by the tour guides to dance in return for thrown bananas and biscuits [9].

Apart from this degrading treatment, the few remaining Andaman Islanders are under constant threat of infectious disease, and vulnerable to the effects of poaching and deforestation on their traditional ways of living [10]. After so many millennia of survival in their Indian Ocean outpost, their existence is precarious; they deserve to be left alone.
